# DGEMRIC in the Assessment of Pre-Morphological Cartilage Degeneration in Rheumatic Disease: Rheumatoid Arthritis vs. Psoriatic Arthritis

**DOI:** 10.3390/diagnostics11020147

**Published:** 2021-01-20

**Authors:** Daniel B. Abrar, Christoph Schleich, Miriam Frenken, Stefan Vordenbäumen, Jutta Richter, Matthias Schneider, Benedikt Ostendorf, Sven Nebelung, Philipp Sewerin

**Affiliations:** 1 Medical Faculty, Department of Diagnostic and Interventional Radiology, University Dusseldorf, 40225 Düsseldorf, Germany; Christoph.Schleich@med.uni-duesseldorf.de (C.S.); Miriam.Frenken@med.uni-duesseldorf.de (M.F.); sven.nebelung@med.uni-duesseldorf.de (S.N.); 2 Department and Hiller Research Unit for Rheumatology, Heinrich Heine University Düsseldorf, Moorenstrasse 5, 40225 Düsseldorf, Germany; Stefan.Vordenbaeumen@med.uni-duesseldorf.de (S.V.); jutta.richter@med.uni-duesseldorf.de (J.R.); matthias.schneider@med.uni-duesseldorf.de (M.S.); ostendorf@rheumatologie-am-hofgarten.de (B.O.); philipp.sewerin@med.uni-duesseldorf.de (P.S.)

**Keywords:** psoriatic arthritis, rheumatoid arthritis, cartilage, magnetic resonance imaging, dGEMRIC, compositional imaging

## Abstract

Background: Even though cartilage loss is a known feature of psoriatic (PsA) and rheumatoid arthritis (RA), research is sparse on its role in the pathogenesis of PsA, its potential use for disease monitoring and for differentiation from RA. We therefore assessed the use of delayed gadolinium-enhanced magnetic resonance imaging of cartilage (dGEMRIC) to evaluate biochemical cartilage changes in metacarpophalangeal (MCP) and proximal interphalangeal (PIP) joints in PsA patients and compared these to RA patients. Materials and Methods: A total of 17 patients with active PsA and 20 patients with active RA were evaluated by high-resolution 3 Tesla dGEMRIC using a dedicated 16-channel hand coil. Images were analyzed by two independent raters for dGEMRIC indices and joint space width (JSW) at MCP and PIP joint levels. Results: No significant differences of dGEMRIC values could be found between both study populations (PsA 472.25 ms, RA 461.11 ms; *p* = 0.763). In all RA and most PsA patients, PIP joints showed significantly lower dGEMRIC indices than MCP joints (RA: D2: *p* = 0.009, D3: *p* = 0.008, D4: *p* = 0.002, D5: *p* = 0.002; PsA: D3: *p* = 0.001, D4: *p* = 0.004). Most joint spaces had similar widths in both disease entities and no significant differences were found. Conclusions: As evaluated by dGEMRIC, the molecular composition of the MCP and PIP joint cartilage of PsA patients is similar to that of RA patients, demonstrating the scientific and clinical feasibility of compositional magnetic resonance (MR) imaging in these disease entities. Patterns and severity of compositional cartilage degradation of the finger joints may therefore be assessed beyond mere morphology in PsA and RA patients.

## 1. Introduction

Cartilage damage and bone destruction are the main features of progressive rheumatoid arthritis (RA) [1,2]. Even though the exact pathogenesis of rheumatoid arthritis (RA) is subject to ongoing research, three main patho-mechanisms are considered to eventually lead to cartilage destruction: a. synovial inflammation leading to secondary infiltration and destruction of bone and cartilage (outside-in-model) [3]; b. primary inflammation of subchondral bone marrow that secondarily involves cortical bone and cartilage [3,4,5]; c. primary affection of joint cartilage by deposition of immune complexes [6]. Eventually, all mechanisms lead to functional disability of joints, wherein cartilage damage is considered to be pivotal [7,8]. Unlike RA, the research on cartilage damage in psoriatic arthritis (PsA) is sparse, even though it is a known feature of the disease [9]. In particular, studies evaluating biomarkers such as cartilage oligo-matrix protein (COMP) and osteo-protegerin (OPG) could demonstrate an involvement of cartilage in PsA that was not limited to late stages of the disease [10,11,12,13]. Research has also shown that bone and cartilage damage in RA is frequently associated with entheseal insertion sites due to mechanical stress and spreading inflammation [14]. Considering that PsA is an entheseal disease, driven by inflammation of the so-called synovio–entheseal complex, an entheseal-driven involvement of bone and cartilage seems evident [15,16].

Early diagnosis and treatment are pivotal for a favorable clinical outcome in both entities, RA and PsA. Hence, treat-to-target strategies have emerged over the past decade [17,18,19]. However, the role of magnetic resonance imaging (MRI) in clinical strategies is less clear. On the one hand, in RA, bone marrow edema and MRI, erosions are highly predictive of future erosions, even though there is only little evidence for this in PsA; on the other hand, a recent trial failed to demonstrate any benefit from an MRI-remission strategy [20]. Thus, more research is clearly needed. Morphological MR imaging is commonly used in clinical contexts and beyond, for example, by the Outcome Measures in RA Clinical Trials (OMERACT) working group for the quantitative assessment of RA and PsA in research studies. Additionally, there are several compositional MRI techniques available that allow the detection of cartilage changes on a molecular level preceding morphological damage [21]. Delayed gadolinium-enhanced MRI of cartilage (dGEMRIC) is the gold-standard technique for compositional cartilage imaging that allows the visualization of proteoglycan depletion in cartilage by analysis of the fixed-charge density [22]. The negatively charged side chains of proteoglycans bring about charge repulsion of equally negatively charged gadolinium molecules after intravenous administration and diffusion into cartilage tissue. Consequently, the concentration of proteoglycans is inversely proportional to the concentration of gadolinium; dGEMRIC is considered the best validated and most robust technique for quantifying proteoglycan content in vivo and in clinical trials [23].

Using dGEMRIC, several studies have shown a strong correlation of local joint inflammation and early cartilage alterations in RA [24,25]. However, to the best of our knowledge, no such data exists for PsA patients. Accordingly, the aim of our study was to assess articular cartilage composition by dGEMRIC across a cohort of PsA patients as compared to RA patients and to evaluate both patterns and severity of compositional (i.e., pre-morphological) cartilage degradation in both disease entities. Our study’s hypotheses were a) patterns and severity of compositional cartilage degradation of the finger joints could be assessed by dGEMRIC in PsA patients; and b) these were significantly different in PsA patients as compared to RA patients.

## 2. Materials and Methods

### 2.1. Study Population

21 patients with PsA (mean age 47 ± 6, range 26–72 years, male/female 11/10) fulfilling the classification criteria for psoriatic arthritis (CASPAR), mean disease duration 4 ± 3.6 years and suffering from peripheral joint involvement and dactylitis were prospectively recruited for the “Analysis of the DActylic Melange” (ADAM) research initiative [26]. All patients had failed methotrexate (MTX) monotherapy and were escalated to Etanercept (Enbrel^®^ 50 mg s.c. fortnightly, Pfizer). 17 patients (mean age 53.7 ± 11.6; minimum/maximum 26/72 years, male/female 9/8) were included, and four patients did not want to participate in the study.

20 patients (mean age 46 ± 15.7, range 19–67 years, male/female 9/11) fulfilling the American College of Rheumatology (ACR)/European League Against Rheumatism (EULAR) 2010 criteria for RA with a mean disease duration <6 months (mean duration 8 weeks, range 2–22 weeks) from the ‘Cartilage in early RA’ (CAR-ERA) study were similarly included. Patients maintained either MTX monotherapy or received a combination of MTX and adalimumab. Patients were blinded for their therapy regime.

The study was approved by the local ethics committee (Ethics committee of the medical faculty of the Heinrich-Heine-University Dusseldorf, 4962R, MO-LKP-719, approved on 01 April 2015 and 17 September 2015). Written and informed consent was obtained from all patients before initiation of the study. Patient recruitment and consecutive MRI measurements were performed between 01/2015 and 12/2017.

### 2.2. MR Imaging

For all patients, we used a clinical 3T MRI scanner (Magentom Skyra, Siemens Healthineers, Erlangen, Germany) and a receive-only 16-channel hand coil (3T Tim Coil, Siemens).

All patients were scanned in the prone position with the clinically dominant hand extended overhead, palm facing down (‘superman position’).

The morphologic MRI protocols were designed according to the recommendations of the OMERACT working group for PsA and RA [27,28].

In practical terms, the imaging protocol included pre- and post-contrast coronal T1-weighted turbo spin echo (TSE) sequences before and after intravenous administration of a gadolinium-based contrast agent (Gd-DOTA-], Dotarem, Guerbet Villepinte, France) in a double dose, i.e., 0.4 mmoll/kg bodyweight). Additionally, post-contrast fat-saturated T1-weighted sequences in at least two different orthogonal planes and non-contrast enhanced, fat-saturated T2-weighted/short tau inversion recovery (STIR) sequences were acquired. Further, two 3D fast low angle shot (3D-FLASH) sequences using two different excitation flip angles (5° and 26°) were obtained for T1 mapping.

For the entire study population, compositional MRI using the dGEMRIC technique of the MCP and PIP joints of fingers 2–5 was conducted 40 min after intravenous injection of Gd-DOTA-. We used a flip-angle three-dimensional gradient-echo imaging (FLASH) sequence with two excitation flip angles (5° and 26°). 40 sagittal slices were obtained perpendicular to each joint’s surface. The total acquisition time was approximately 2 min 25 sec. The detailed sequence parameters were as follows: coronal T1 TSE (TR/TE in ms, PsA: 862/27, RA: 25/860; flip angle in °, PsA: 150, RA: 150; slice thickness in mm, PsA: 2.5, RA: 2.5; field of view in mm, PsA: 140, RA: 120), coronal STIR (TR/TE in ms, PsA: 5560/31, RA: 31/5560; flip angle in °, PsA: 120, RA: 120; slice thickness in mm, PsA: 2.5, RA: 2.5; field of view in mm, PsA: 140, RA: 120), transversal T1 SE fat-saturated after iv contrast (TR/TE in ms, PsA: 807/16, RA: 807/16; flip angle in °, PsA: 90, RA: 90; slice thickness in mm, PsA: 3.0, RA 3.0; field of view in mm, PsA: 130, RA: 130), coronal T1 TSE after iv contrast (TR/TE in ms, PsA: 862/27, RA: 25/120; flip angle in °, PsA: 150, RA; 150; slice thickness in mm, PsA: 2.5, RA: 2.5; field of view in mm, PsA: 140, RA: 120), flip-angle three-dimensional gradient-echo imaging (FLASH, TR/TE in ms, PsA: 5.8/1.9, RA: 5.8/1.9; flip angle in °, PsA: 8/26, RA: 8/26; slice thickness in mm, PsA: 3.0, RA: 3.0; field of view in mm, PsA: 140, RA: 140, imaging matrix in PsA and RA 312 × 384).

### 2.3. Image Analysis

For the quantitative analysis of joint space widths (JSW), T1-weighted images obtained perpendicularly to the joint surfaces were used. Following the approach of Herz et al. [29] and using the caliper tool of the in-house picture archiving and communication system (PACS, IDS 7, Sectra AB, Linköping, Sweden), JSW was measured in mm at each radial, ulnar and central aspect of the joint as the distance between the proximal and distal cortical bone. For compositional analyses of cartilage quality with dGEMRIC, motion correction was performed using STROKETOOL (Digital Image Solutions, Frechen, Germany) for all images to reduce movement artifacts. This tool has been validated for dGEMRIC analyses of the finger joints and corrects for patient motion between the measurements using a dedicated image registration method [30].

T1 maps were analyzed using region of interest (ROI) measurements to evaluate biochemical cartilage quality. T1 values were calculated per pixel. Gradient-echo (GE) images with a flip angle of 5° were applied for anatomical identification of articular cartilage. ROI were drawn into the proximal and distal portion of the articular cartilage of MPC and PIP joints 2–5 on a single sagittal slice. Readers were allowed to adjust the window settings as required to guarantee optimal visualization of ROI. After ROI placement a second reader confirmed the optimal placement before the ROI were transferred to a simultaneously registered T1 map. DGEMRIC indices were recorded in ms. All images were analyzed by two readers (DBA and CS, radiologists trained in musculoskeletal imaging with three and eight years of experience) who were blinded for patients’ data; in case of different findings, the analysts decided by common agreement. For JSW and dGEMRIC measurements, mean values were calculated.

### 2.4. Statistical Analysis

All statistical analyses were performed using SPSS software (IBM, version 22, Armonk, NY, USA) by DBA. For descriptive analysis, mean, standard deviation, minimum and maximum values were calculated. Datasets were tested for normality by Kolmogorov-Smirnov test with Lilliefors significance correction. Means were compared by student’s t-test. *p*-values < 0.05 were considered to be significant.

## 3. Results

The descriptive analysis (mean, standard deviation and range) of dGEMRIC values at MCP and PIP joints in both study populations is displayed in Table 1. Mean dGEMRIC indices at the MCP joint level D2-5 ranged from 516.2 to 552.1 ms in PsA patients and from 519.3 to 575.8 ms in RA patients. At the PIP joint level D2-5 mean dGEMRIC indices ranged from 338.6 to 450.0 ms in PsA patients and from 399.5 to 439.4 ms in RA patients, respectively. By trend, dGEMRIC indices tended to be higher in PsA patients than in RA patients, yet these differences were not significant (Table 1).

JSW of MCP and PIP joints of both study populations are presented in Table 2. Only PIP joints 2 and 3 showed significantly wider joint spaces in PsA than in RA patients (*p* = 0.005). All other joints displayed no significant disease-related differences in JSW.

The comparative analysis of dGEMRIC indices of PsA and RA patients is illustrated in Table 3. There was no significant difference between the mean dGEMRIC indices of PsA and RA patients, neither at the MCP nor at the PIP joint level.

**Figure 1:**
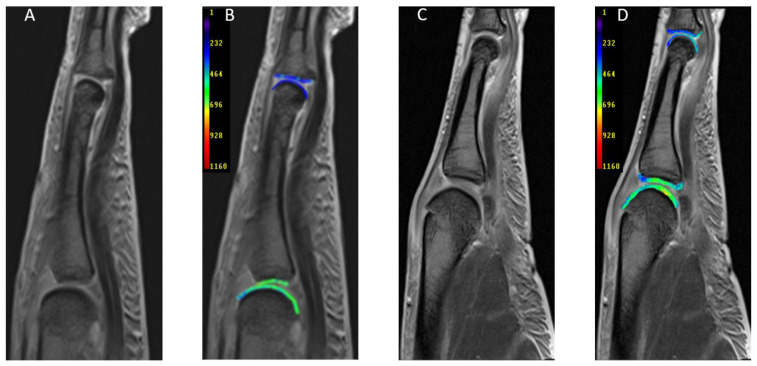
Representative sagittal magnetic resonance (MR) images of MCP and PIP joints of the 2nd digit of patients suffering from PsA (**A**,**B**) and RA (**C**,**D**). (**A**,**C**) give the morphological T1 maps, while (**B**,**D**) give the corresponding dGEMRIC maps of the same patients. 62-year-old male with PsA (**A**,**B**) and 57-year-old female with RA (**C**,**D**).

The comparative analysis of dGEMRIC indices of MCP and PIP joints within each study population is shown in Table 4 In RA patients we found significantly lower dGEMRIC indices at PIP than at MCP joints (D2: *p* = 0.009; D3: *p* = 0.008; D4: *p* = 0.002; D5: *p* = 0.002). This finding was partly reflected in PsA patients, as their PIP joints 3 and 4 showed significantly lower dGEMRIC indices than the corresponding MCP joints (D3: *p* = 0.001; D4: *p* = 0.004). Representative dGEMRIC maps are visualized in Figure 1.

## 4. Discussion

The most important finding of this study is that the degree of proteoglycan loss at the MCP and PIP joints (as assessed by dGEMRIC) is not significantly different between PsA and RA patients.

RA and PsA are chronic inflammatory disorders that cause bone and cartilage destruction and eventually lead to joint mutilation and functional disability. Albeit cartilage damage has been investigated both in terms of morphology and composition for RA, it has not yet been the focus of research in PsA. As imaging biomarkers that allow a more clear-cut differentiation of RA, PsA and other inflammatory and non-inflammatory joint diseases such as osteoarthritis are still lacking, we set out to introduce dGEMRIC for the evaluation of proteoglycan loss in PsA patients and to compare the degree of early cartilage loss to RA patients [31,32].

According to our findings the degree of proteoglycan loss at MCP and PIP joints is equally distributed in both, PsA and RA patients. Even though cartilage damage is a known feature in PsA, research is sparse on its role in pathogenesis and it is widely considered a late feature of the disease, especially as compared to RA [33,34,35]. Cartilage loss in RA, on the other hand, has been the focus of intense research over the last decades and has helped to further delineate the association between cartilage damage and joint inflammation [6]. Even though the question of whether cartilage damage is the reason for, or result of, inflammation remains open, unequivocal evidence suggests that disease activity is closely related to cartilage damage [6]. In the context of our study, a higher degree of early cartilage degradation could have been assumed in RA patients. Similarly, early cartilage loss in PsA could be of significance to the pathogenesis of the disease and provide a potential diagnostic tool beyond the initial diagnosis, i.e., also for disease monitoring, as it is in RA [27]. Therefore, future studies should investigate the association of early cartilage changes detected by MRI and clinical inflammation as well as serum biomarkers in patients with PsA.

Despite roughly similar dGEMRIC indices in RA and PsA patients, we found significantly lower dGEMRIC values, indicating a higher degree of proteoglycan loss in PIP than in MCP joints in both entities. Even though not included in the OMERACT RA MRI score (RAMRIS), it is long known that RA commonly and severely affects PIP next to MCP and wrist joints [27,36]. PsA, on the other hand, is a more heterogeneous disease that is traditionally divided into five subtypes and can affect various joints, one of them commonly being the PIP joint. This explains why PIP joints are included in the OMCERACT PsAMRIS [28]. It is known that RA and PsA share pathophysiological features and that a distinction between the two can be difficult, especially in cases of symmetrical joint involvement [31]. Our findings of equal dGEMRIC values in both entities with a predominant proteoglycan loss of the PIP joint cartilage confirm the known dilemma of the potentially difficult differentiation between the two disease entities.

Generalizability of the study is hampered by a few limitations. Firstly, we used a small study population. Therefore, further research with a larger sample size is needed. Nonetheless, comparative evaluation was rendered feasible by the application of consistent imaging protocols and strict inclusion criteria for both disease entities. Second, some patients of our study population were older than 60 years of age. Therefore, coexisting osteoarthritis might have been a confounding factor. Third, the mean disease duration of the PsA and RA study population differed by approximately 192 weeks (RA 8, PsA 200 weeks). Applying current definitions, we hence compared “non-early” PsA to “early” RA populations (“early” RA: disease duration <12 months; “early” PsA: disease duration <24 months) [37,38]. Thus, the comparability of both populations is potentially limited in this respect. That is why, future studies using more strictly defined study cohorts (e.g., “early” RA versus “early” PsA) in the assessment of compositional MRI techniques need to corroborate our findings, especially if a distinction between both entities was the aim. In addition, as absolute dGEMRIC indices vary among different studies and protocols, inter-study comparability is limited, and absolute values cannot be readily translated. Fourth, we used Magnevist for our dGEMRIC technique in RA patients since it is the best validated technique for assessment of proteoglycans [39]. However, the European Medical Association (EMA) banned Magnevist due to its potential complications, e.g., cerebral and cerebellar gadolinium depositions [40]. That is why we applied a different, non-linear contrast agent (Dotarem) for the PsA study group, which has been used in prior studies for dGEMRIC [41]. Due to the ban on linear gadolinium-based contrast agents, future studies need to be based on gadolinium-based contrast agents that are potentially less harmful.

## 5. Conclusions

In conclusion, the molecular cartilage composition at MCP and PIP joints of “non-early” PsA patients is similar to that of a control group of “early” RA patients. Hence, biochemical compositional imaging on the basis of dGEMRIC could be of value for disease monitoring in PsA patients, as it is in RA. This study demonstrates scientific and clinical feasibility of compositional MR imaging in PsA and RA patients, thereby providing a potential framework for more elaborate assessment of patterns and severity of compositional cartilage degradation of the finger joints beyond mere morphology. Prospectively, compositional MR imaging may be applied in the context of diagnostic differentiation and assessment of both disease entities and other inflammatory and non-inflammatory joint disorders, as well as in the monitoring of disease activity under treatment.

## Figures and Tables

**Table 1:** Descriptive analysis (mean values, standard deviations (SD) and range (minimum/maximum)) of delayed gadolinium-enhanced magnetic resonance imaging of cartilage (dGEMRIC) indices [ms] of metacarpophalangeal (MCP) and proximal interphalangeal (PIP) joints D2-5 in psoriatic arthritis (PsA) and rheumatoid arthritis (RA) patients.

dGEMRIC [ms]	D2		D3		D4		D5	
	MCP	PIP	MCP	PIP	MCP	PIP	MCP	PIP
PsA								
Mean	523.68	420.81	516.2	338.62	552.07	407.88	537.35	450.01
SD	180.69	113.73	147.05	98.52	123.11	125.44	143.22	118.0
Minimum	272.0	202.9	259.3	190.6	348.3	235.5	327.3	277.2
Maximum	951.4	639.6	717.0	49.4	806.4	678.7	861.9	674.3
RA								
Mean	519.27	399.53	545.12	415.59	576.81	439.39	537.52	435.97
SD	106.11	146.95	117.0	139.65	126.65	106.33	99.82	85.97
Minimum	349.1	225.6	335.5	249.2	298.7	312.6	366.3	307.8
Maximum	679.6	777.9	764.9	671.2	834.1	644.3	739.3	607.8

**Table 2:** Descriptive and comparative analysis of joint space width (JSW) (mm) of MCP and PIP 2–5 in RA and PsA patients. *p*-values < 0.05 are considered significant and are given in bold type.

JSW [mm]	D2		D3		D4		D5	
	PsA	RA	PsA	RA	PsA	RA	PsA	RA
MCP								
Mean	1.59	1.65	1.57	1.44	1.34	1.31	1.44	1.35
SD	0.19	0.33	0.26	0.22	0.24	0.24	0.23	0.26
Minimum	1.30	1.13	1.15	1.13	0.92	1.02	1.08	0.98
Maximum	1.96	2.55	2.03	1.86	1.80	1.90	1.80	1.88
*p*-value	0.536		0.109		0.640		0.271	
PIP								
Mean	1.09	0.88	1.14	0.88	0.95	0.85	0.90	0.82
SD	0.33	0.14	0.30	0.17	0.35	0.16	0.30	0.24
Minimum	0.68	0.56	0.66	0.65	0.54	0.59	.049	0.54
Maximum	1.72	1.12	1.62	1.19	1.75	1.17	1.59	1.59
*p*-value	**0.005**		**0.005**		0.272		0.364	

**Table 3:** Mean differences in dGEMRIC indices [ms] at MCP and PIP joints D2-5 comparing PsA and RA patients. Positive differences indicate higher dGEMRIC values in PsA than RA patients for the specific finger joint, and vice versa. CI: confidence interval.

dGEMRIC [ms] PsA vs. RA	D2		D3		D4		D5	
	MCP	PIP	MCP	PIP	MCP	PIP	MCP	PIP
Mean difference	4.41	21.28	−28.92	−76.97	−24.74	−31.51	−0.17	14.03
95% CI lower limit	−98.71	−74.57	−124.16	−168.54	−113.91	−116.74	−86.7	−165.89
95% CI upper limit	107.54	117.14	66.33	14.6	64.43	53.72	86.36	129.04
*p*-value	0.931	0.653	0.540	0.096	0.576	0.456	0.997	0.798

**Table 4:** dGEMRIC values of PsA and RA patients comparing MCP and PIP joints D2–5. CI: confidence interval. *p*-values < 0.05 were considered to be significant and are given in bold type.

dGEMRIC. MCP vs. PIP	D2		D3		D4		D5	
	PsA	RA	PsA	RA	PsA	RA	PsA	RA
Mean difference	102.87	119.74	177.58	129.53	144.19	137.41	87.34	101.54
95% CI lower limit	−10.05	31.97	82.82	36.88	51.23	55.13	−13.06	38.44
95% CI upper limit	215.79	207.51	272.34	222.18	237.15	219.69	187.74	164.65
*p*-value	0.073	**0.009**	**0.001**	**0.008**	**0.004**	**0.002**	0.086	**0.002**

## Data Availability

Data can be obtained from the authors upon reasonable request.
